# Evaluation of Immunohistochemical Expression of Enhancer of Zeste Homolog 2 (EZH2) and Its Association With Clinicopathological Variables in Carcinoma Cervix

**DOI:** 10.7759/cureus.36946

**Published:** 2023-03-31

**Authors:** Aditi Priya, Jai K Chaurasia, Pushpalatha K, Hemlata Panwar, Shakti K Yadav, Neelkamal Kapoor

**Affiliations:** 1 Pathology and Laboratory Medicine, All India Institute of Medical Sciences, Bhopal, Bhopal, IND; 2 Obstetrics and Gynaecology, All India Institute of Medical Sciences, Bhopal, Bhopal, IND; 3 Pathology and Laboratory Medicine, AIl India Institute of Medical Sciences, Bhopal, Bhopal, IND

**Keywords:** clinicopathological, cervix, carcinoma, immunohistochemical, enhancer of zeste homolog 2

## Abstract

Introduction: Carcinoma cervix is the fourth most common cancer worldwide and is one of the leading causes of cancer death in women. Recently, immunohistochemical expression of biomarkers has been utilized as indicators of disease progression, aggressiveness for predicting the prognosis in various cancers. DNA methylation of genes plays an important role in pathogenesis of carcinoma cervix and detection of aberrant methylation can be utilized for detection of carcinoma cervix and monitoring of its progression. Enhancer of Zeste Homolog 2 (EZH2) is a histone methyltransferase and catalyzes methylation of histone H3 and plays an important role in tumor cell proliferation, invasion, and metastasis. The aim of this study was to analyze the pattern, distribution, and grade of immunohistochemical expression of EZH2 in carcinoma cervix and study its association with clinico-pathological variables such as age, site and size of tumor, type of growth, tumor grade, histological subtype, lymph node metastasis, and stage of the tumor according to the Federation of Gynaecology and Obstetrics (FIGO).

Materials and methods: This observational study was carried out in the Department of Pathology & Lab Medicine, at our institute. A total of 60 consecutive histopathologically confirmed cases of carcinoma cervix from January 2018 to June 2022 were subjected to immunohistochemistry (IHC) for EZH2. The immunohistochemical score for each case was obtained by multiplying the intensity and percentage of positive cells for EZH2. An immunohistochemical score of four or greater than four was considered as high immunoexpression. The immunohistochemical results were correlated with clinico-pathological variables.

Results: The data were analyzed using relevant statistical methods using SPSS version 23 (IBM Corp., Armonk, NY). To find the significant difference (p value) and association, chi-square test along with Pearson chi-square were used, wherever necessary. A p value of <0.05 was considered as significant. High immunoexpreesion of EZH2 exhibited a significant association (p < 0.05) with the tumor grade, histologic subtype, lymphnode metastasis, and FIGO stage.

Conclusions: The results of our study affirm that a significant association exists between immunohistochemical expression of EZH2 with tumor grade, histological subtype, lymphnode metastasis, and FIGO stage which can be utilized in future studies with larger sample size to further strengthen the association of EZH2 immunoexpression in cancer cervix patients that may aid in the development of the targeted therapy in near future.

## Introduction

Carcinoma cervix is the fourth most common cancer worldwide and is one of the leading causes of cancer death in women [1-2]. In India, it alone bears 23% of the global cervical cancer burden and is also the leading cause of morbidity and mortality [3]. Early-stage cervical cancers are amendable to surgery while cancers with distant metastasis or recurrence are mostly fatal. Moreover, most of the cases of carcinoma cervix present in advanced stages result in poor prognosis and increased mortality. Despite the standardized treatment of cancer cervix, prediction of patient’s outcome remains a challenge [4]. Recent requirement of personalized treatment has led to the development of ‘Biomarkers’ which are being utilized for targeted therapy in various cancers [5]. Immunohistochemical expression of biomarkers can be utilized as indicators of disease progression, aggressiveness and can predict prognosis in carcinoma cervix, thereby reducing mortality [6].

DNA methylation of genes plays an important role in the development and progression of cervical cancer. Detection of aberrant methylation can be utilized for detection and monitoring of progression of carcinoma which suggests that host gene methylation analysis may be a crucial tool for development of new targeted treatment therapies and for guiding treatment [7].

Enhancer of Zeste Homolog 2 (EZH2) is a histone methyltransferase and catalyses trimethylation of histone H3 at lysine 27 (H3K27) leading to DNA methylation, chromatin remodeling, and silencing of tumor suppressor genes [8-10]. It has oncogenic activity and is shown to inhibit cell differentiation and induces epithelial mesenchymal transition (EMT) directly via histone trimethylation. It plays important role in tumor cell proliferation, invasion, and metastasis [6, 11]. The role of EZH2 has been documented in pathogenesis of various carcinomas such as breast, lung, thyroid, and endometrial cancers [12-15]. However, its role in pathogenesis of cervical cancer is less explored. The aim of this study was to analyze the pattern, distribution, and grade of immunohistochemical expression of EZH2 in carcinoma cervix and correlate it with clinico-pathological variables such as age of patient, size and site the tumor, type of growth, tumor grade, histological subtype, lymph node metastasis, and stage of the tumor according to the Federation of Gynaecology and Obstetrics (FIGO).

## Materials and methods

This observational study was carried out in the Department of Pathology & Lab Medicine, at our institute after the approval from Institutional Human Ethics Committee (IHEC). A total of 60 consecutive cases of carcinoma cervix over a period from January 2018 to June 2022 were included. The histopathology, demographic, and clinical details of the patients were retrieved from the histopathology requisition form and from the medical records. The details of gross findings including site and size of the tumor, type of growth along with FIGO stage were noted from histopathology and clinical medical records. Hematoxylin and Eosin (H&E) stained slides were retrieved for noting microscopic findings including histological subtype, tumor grade, and lymph node status. A list of cases fulfilling the inclusion criteria was prepared. Paraffin blocks of the selected cases were then retrieved for obtaining sections for performing IHC for EZH2.

Inclusion criteria

Only histopathological confirmed cases of carcinoma cervix were included.

Exclusion criteria

**Figure 1:**
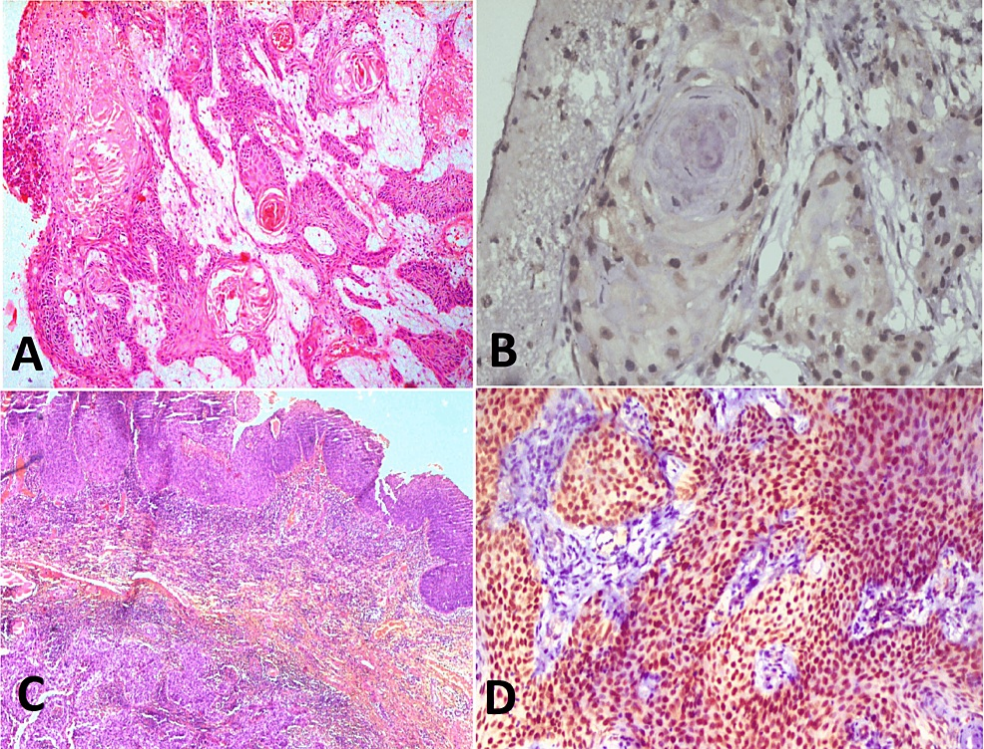
A: Showing Grade 1 (well differentiated) keratinizing SCC with keratin pearls (H&E x10). B: Corresponding IHC in the same case showing weak nuclear positivity of EZH2 (Score 3) (IHC EZH2 x40). C: Showing invasive growth of Grade 2 (moderately differentiated) non-keratinizing SCC (arrow) (H&E x4). D: Corresponding IHC in the same case showing strong nuclear positivity of EZH2 (Score 9) (IHC EZH2 x40). SCC, squamous cell carcinoma; IHC, immunohistochemistry

**Figure 2:**
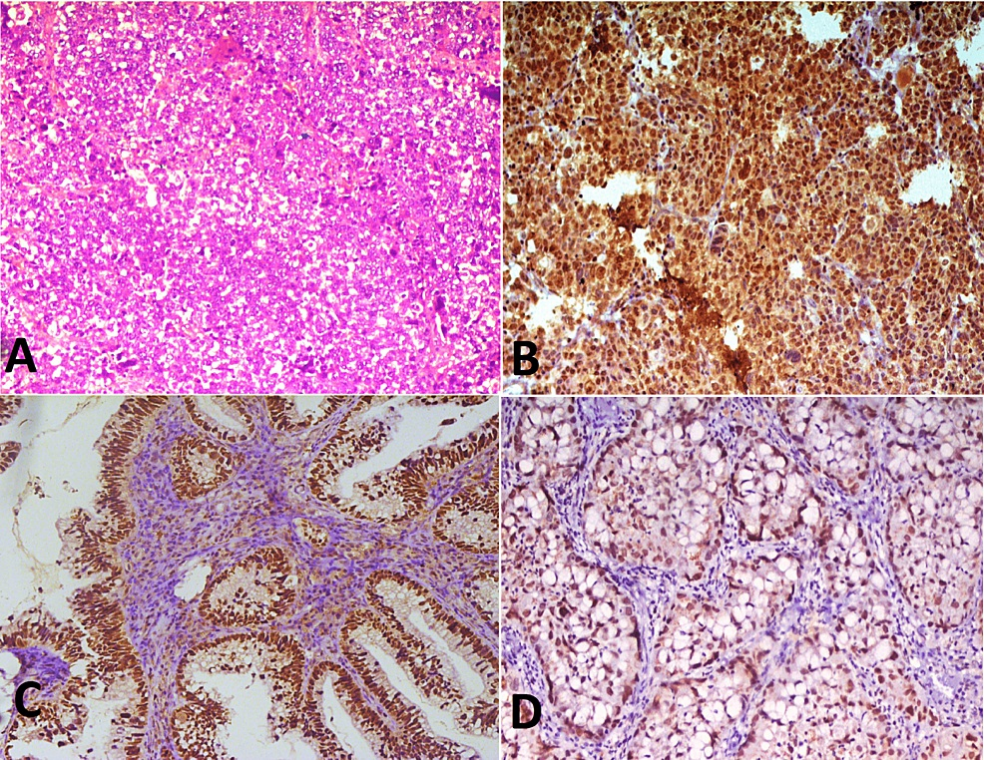
A: Showing Grade 3 (poorly differentiated) SCC (H&E x20). B: Corresponding IHC showing strong nuclear positivity of EZH2 (Score 9) (IHC EZH2 x20). C: Showing strong nuclear positivity of EZH2 (Score 9) in adenocarcinoma, usual type (IHC EZH2 x20). D: showing moderate nuclear positivity of EZH2 (Score 6) in adenocarcinoma, gastric type (IHC EZH2 x20). SCC, squamous cell carcinoma; IHC, immunohistochemistry; EZH2, enhancer of Zeste homolog 2

Cases with inadequate representative material in the archival blocks or unavailable block were excluded. The carcinoma cervix cases were graded into Grade 1 (well differentiated) (Figure 1A), Grade 2 (moderately differentiated) (Figure 1C), and Grade 3 (poorly differentiated) (Figure 2A) based on microscopic examination of H&E stained slides.

 

Following histological subtypes of carcinoma cervix were encountered and were included in the study:

1. Keratinizing squamous cell carcinoma (SCC)

2. Non-keratinizing SCC

3. Basaloid SCC

4. Papillary SCC

5. Lymphoepithelioma-like carcinoma

6. Adenocarcinoma, Usual type

7. Adenocarcinoma, Gastric type

8. Adenocarcinoma, Clear cell type

Immunohistochemistry

Immunohistochemistry for EZH2 was performed on the selected cases fulfilling the inclusion criteria. For this, appropriate paraffin embedded blocks of cases were selected and four-micron thick sections were obtained on hydrophobic charged slides. IHC was carried out using the commercially available EZH2 rabbit polyclonal antibody, (pack size 100 µL, concentration of 1 mg/mL) (Clone AF5150#1486) from Affinity Biosciences, Cincinnati, OH, USA used in 1:100 dilution on the sections using Ventana benchmark XT automated IHC stainer. The positive and negative controls were also taken for IHC. Positive controls were taken from commercially available tissue with standardized and documented IHC results. Negative control included non-cancerous adjacent cervical tissue which served as internal negative control.

Interpretation of immunohistochemistry

The immunohistochemical expression of EZH2 was considered positive by presence of brown nuclear staining (Figure 1B,C). The interpretation of IHC of EZH2 was performed by studying the pattern, distribution, and grade of the immunohistochemical expression. The score of staining intensity and the score of percentage of positive cells were assessed as follows:

Intensity Scoring:

Score 0 - Absence of staining

Score 1 - Weak nuclear staining

Score 2 - Moderate nuclear staining

Score 3 - Intense nuclear staining

Scoring of percentage of positive cells:

Score 1 - 0-33%

Score 2 - 34%-66%

Score 3 - > 67%

A final staining score for EZH2 was then obtained by multiplying the score of staining intensity and the percentage of positive cells. An immunohistochemical score of four or greater than four (≥ 4) was considered as high immunoexpression and a score of less than four was considered as low immunoexpression (Table 1) [6].

**Table 1:** Distribution of cases according to intensity score, percentage of positive cells, and final immunohistochemical expression score of EZH2. EZH2, Enhancer of Zeste homolog 2

	Immunohistochemical expression of EZH2								
	Intensity grading score				Percentage of positive cells			Total score	
	Score 0	Score 1	Score 2	Score 3	Score 1 (0-33%)	Score 2 (34%-66%)	Score 3 (>67%)	Low (<4)	High (≥4)
Number of cases	0 (0%)	19 (31.7%)	15 (25%)	26 (43.3%)	00 (0%)	04 (6.7%)	56 (93.3%)	19 (31.7%)	41 (68.3%)

Clinicopathological correlation

Immunohistochemical expression of EZH2 was correlated with clinico-pathological features such as age of the patient, size and site of tumor site, type of growth, tumor grade, histological subtype, lymph node metastasis, and FIGO staging (Table 2).

**Table 2:** Association of immunohistochemical expression of EZH2 with various clinicopathological variables. SCC, squamous cell carcinoma; EZH2, enhancer of Zeste homolog 2

	Number of cases	Immunohistochemical expression of EZH2	
		High	Low
Age (in years)				
≤50	29	20	9
>50	31	20	11
Size (in cm)				
≤ 4	29	17	12
>4	31	24	7
Tumor Grade				
Grade 1	8	1	7
Grade 2	50	38	12
Grade 3	2	2	0
FIGO Stage				
I	17	7	10
II	28	21	7
III	13	11	2
IV	2	2	0
Histological Subtypes			
Keratinizing SCC	6	1	5
Non-keratinizing SCC	40	30	10
Papillary SCC	5	5	0
Basaloid SCC	3	2	1
Lymphoepithelioma-like carcinoma	1	0	1
Adenocarcinoma, Usual Type	3	2	1
Adenocarcinoma, Gastric Type	1	1	0
Adenocarcinoma, Clear Cell Type	1	0	1
Lymph Node Metastasis				
Present	21	20	01
Absent	39	21	18

Statistical analysis

The collected data were analyzed using SPSS statistics software 23.0 Version. To describe the data, descriptive statistics, frequency analysis, and percentage analysis were used for categorical variables and the mean and standard deviation (SD) were obtained for continuous variables. To find the significant difference (p value) and correlation, chi-Square test along with Pearson chi-Square were used, wherever necessary. The probability value p < 0.05 was considered as significant.

## Results

Out of the 60 cases, 41 cases (68.3%) showed high immunoexpression of EZH2 (score ≥ 4) while 19 cases (31.7%) showed low immunoexpression (score < 4) of EZH2 (Table 1).

Age

The mean age of patients in our study was 52.50 years with age ranging from 23 years to 78 years. There were 29 patients with age ≤ 50 years and 31 patients with age > 50 years. However, there was no statistically significant association of EZH2 (p = 0.511) immunoexpression with age of patients (Table 2).

Signs and symptoms

Majority of patients in the study presented with post-menopausal bleeding (48.0%), followed by discharge per vaginum (24.7%), bleeding per vaginum (16.9%), growth on cervix (6.5%), and pain lower abdomen (3.9%). These signs and symptoms were present for a minimum 15 days duration and maximum duration of 5 years. The mean duration was 9.42 months.

Size of the tumor

Maximum and minimum size of the tumor were 9.2 cm and 0.5 cm respectively. The mean size of the tumor was 4.108 cm. High expression of EZH2 was observed in 77.4% (24/31) cases with tumor size > 4 cm while low expression was noted in 41.37% (12/29) cases with tumor size ≤ 4 cm which was statistically insignificant (p = 0.118) (Table 2).

Site of tumor

Majority of the tumors involved both anterior and posterior lip (84.7%) followed by (13.6%) cases involving only anterior lip and 1.7% cases involving only posterior lip. However, in the present study, no statistically significant association between the EZH2 immunoexpression with site (p = 0.259).

Type of growth

Clinical examination and gross findings revealed that majority of cases had ulceroproliferative growth (54/60, 90%), followed by fungating (2/60, 3.3%), exophytic 1.7% (1/60, 1.7%) and ulcerative 1.7% (1/60, 1.7%) growth. In two (3.3%) cases, no lesion was identified grossly. In the present study, no statistically significant association between the EZH2 immunoexpression with type of growth (p = 0.197) was obtained.

Tumor grade

Majority of the cases belonged to Grade II (50/60, 83.4%) followed by Grade I (8/60, 13.3%) and Grade III (2/60, 3.3%). EZH2 immunoexpression was significantly associated with tumor grade (p = 0.001) (Table 2). High expression of EZH2 was observed in Grade 3 (Poorly differentiated) tumors (2/2, 100%) (Figure 2B) followed by Grade 2 (Moderately differentiated) tumors (39/50, 78%) (Figure 1D) (Table 2). Low expression of EZH2 was observed in Grade I (well differentiated) carcinomas (7/8, 87.5%) (Figure 1B) (Table 2).

FIGO stage

Among the 60 cases, 28 cases (46.7%) were diagnosed FIGO stage II followed by stage I (17/60, 28.3%), stage III (13/60, 21.7%), and stage IV (2/60, 3.3%). High expression of EZH2 was present in 75.0% (21/28) of stage II tumors, 84.6% (11/13) of stage III tumors, and 100% (2/2) of stage IV tumors while low expression of EZH2 was observed predominantly in stage I tumors (10/17, 58.82%) (Table 2). The association of EZH2 immunoexpression was found to be statistically significant (p = 0.031).

Histological subtype

**Figure 3:**
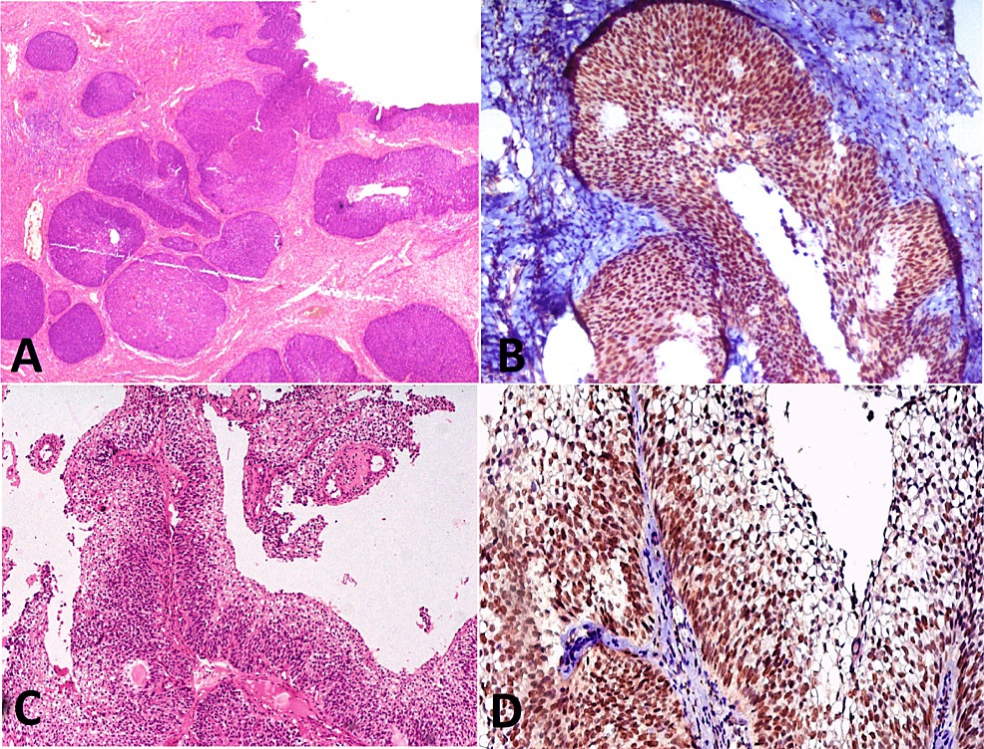
A: Showing invasive nests of basaloid cells in a case of basaloid SCC (H&E x4). B: Corresponding IHC in the same case showing strong nuclear positivity of EZH2 (Score 9) (IHC EZH2 x20). C: Showing papillae with fibrovascular core and lined by squamous epithelium in papillary SCC (H&E x10). D Corresponding IHC showing strong nuclear positivity of EZH2 (Score 9) (IHC EZH2, x20). SCC, squamous cell carcinoma; IHC, immunohistochemistry

Out of 60 cases, non-keratinizing squamous cell carcinoma (SCC) (Figure 1C) was the predominant (40/60, 66.6%) histological subtype, followed by keratinizing SCC (6/60, 10%) (Figure 1A), papillary SCC (5/60, 8.3%) (Figure 3C), basaloid SCC (3/60, 5%) (Figure 3A) and adenocarcinomas, usual type (3/60, 5%) (Table 2). There was one case each of lymphoepithlioma-like carcinoma (1/60, 1.6%), adenocarcinoma, gastric type and adenocarcinoma, clear cell type (Table 2). EZH2 immunoexpression demonstrated significant association with histological subtypes of the tumor (p = 0.018) (Table 2). High expression of EZH2 was most commonly observed in papillary SCC (5/5, 100%) (Figure 3D) non-keratinizing SCC (30/40, 75%) (Figure 1D), basaloid SCC (2/3, 66.6%) (Figure 3B), two cases (2/3, 66.6%) of adenocarcinoma, usual type (Figure 2C) and in one case (1/1, 100%) of adenocarcinoma, gastric type (Figure 2D) while low immunoexpression of EZH2 was predominantly found in keratinizing SCC (5/6, 83.3%) (Figure 1B) and in one case (1/1, 100%) each of adenocarcinoma, clear cell type and lymphoepithelioma-like carcinoma (Table 2).

Lymphnode metastasis

Out of 60 cases, lymphnode metastasis was histologically confirmed in 21 (35%) cases. Out of the 21 cases with lymph node metastasis, high EZH2 immunoexpression was found in 20 (20/21, 95.23%) cases and low immunoexpression in only one case (1/21, 4.7%) (Table 2). The association of EZH2 immunoexpression with lymphnode metastasis was statistically significant (p = 0.025) (Table 2).

## Discussion

Recently the use of biomarkers has been introduced for diagnosis as well as for guiding treatment in various cancers [5]. Immunohistochemical expression of biomarkers such as EZH2 can be utilized as indicators of disease progression, aggressiveness and can predict prognosis in carcinoma cervix [6]. So far, various mechanisms have been postulated by which EZH2 is involved in cervical cancer progression. It is a histone methyltransferase and catalyzes methylation of histone H3 at lysine 27 (H3K27) leading to DNA methylation, chromatin remodeling, and silencing of tumor suppressor genes [8-10]. Thus, it plays an important role in pathophysiology of cancer and has become potential target for cancer therapy. Although, most of the drugs targeting EZH2 for cervical cancer are under clinical trial, however, new EZH2 inhibitor drug such as tazemetostat is approved by FDA for treatment for sarcomas [16].

Other mechanisms by which EZH2 acts is by causing hypermethylation of CpG islands in promoter region of tumor suppressor gene miR-484 by recruiting DNA methyltransferase enzymes. The cell with downregulated miR-484 shows proliferation and undergoes EMT [17]. In an another mechanism, it is postulated that EZH2 activates the Wnt/β-catenin signaling pathway and enhances the cell proliferation in cervical cancer [18]. EZH2 is also identified as a novel factor regulated by the HPV oncogenes protein E6 and E7 in cancer cervix cells. The gene E6/E7 promotes transcriptional activation and expression of EZH2 which further mediates cancer cell proliferation [19].

In the present study, out of 60 cases of carcinoma cervix, high expression of EZH2 was present in 41 cases (68.3%) (Table 1). Similar to our study, Zhang et al. investigated EZH2 expression in 168 cases of cervical SCC and found positive expression rate of 75.6% which was also statistically significant (p < 0.05) [20]. Liu et al. also documented positive expression in 68.3% cases of cervical cancer [21].

The mean age of patients in the present study was 52.50 years which was in concordance with the study conducted by Fathy and Abdelrahman who also documented mean age of 51.22 ± 13.55 [6]. However, the present study did not reveal statistically significant correlation between the age of the patient and EZH2 immunoexpression (p = 0.511) (Table 2). This was in concordance with the previous studies done by Fathy and Abdelrahman, Liu et al., and Jin et al. and who also obtained and documented insignificant association of age and EZH2 immunoexpression with p values of 0.894, 0.476, and 0.46 respectively [6, 21-22].

The tumor size has been documented as an independent risk factor of recurrence and death in carcinoma cervix [23]. In this study, the mean tumor size was 4.108 cm. However, no significant correlation (p = 0.118) was obtained between tumor size and EZH2 immunoexpression (Table 2). This finding was similar to studies by Zhang et al., Jin et al., and Azizmohammadi et al. who also documented insignificant association with p values of 0.849, 0.077, and 0.63 respectively [20, 22, 24].

In this study, among the various prognostically important clinicopathological variables that were analyzed with regard to EZH2 immunoexpression, it was observed that higher FIGO stage of carcinoma cervix showed high immunoexpression score of EZH2 with statistical significance (p = 0.031) (Table 2) which was in concordance with the study done by Zhang et al. who studied 168 cases of cervical SCC of different FIGO stages and observed increasing EZH2 expression with ascending stages of SCC with statistical significance (p < 0.05) [20]. Similarly, in an another study, Chen et al. studied 168 patients with cervical SCC and documented that the expression of EZH2 was upregulated with ascending stages of cervical cancer (p = 0.005) [8]. Azizmohammadi et al. also studied different FIGO stages of cervical cancer including 22 cases (56%) of stage IB-IIA cancer and 17 cases (44%) of stage IIB-IIIA cancer and found statistically significant correlation (p < 0.05) between EZH2 expression and increasing FIGO stages [24]. Thus, immunoexpression of EZH2 in low stage carcinomas can be studied to see any progression to higher stage in advance and this can aid in monitoring the progression and modulating the treatment strategies. The possible explanation of increasing EZH2 immunoexpression with ascending stages of cervical cancer might be that the EZH2 induces EMT directly by repressing the expression of E-cadherin through H3K27 trimethylation. However, in contrast to above studies, Jin et al. studied expression of EZH2 in 117 cases of cervical SCC with different FIGO stages including stage IB1 (85/117), stage 1B2 (10/117), stage IIA1 (8/117), stage IIA2 (8/117), and stage IIB (6/117) and found no statistical correlation between EZH2 expression and FIGO stages (p = 0.100) [22].

In the present study, it was observed that the EZH2 had a significant correlation with histologic subtypes of carcinoma cervix (p = 0.018) (Table 2). High expression of EZH2 was most commonly observed in papillary SCC (5/5, 100%) (Figure 3D), non-keratinizing SCC (30/40, 75%) (Figure 1D), basaloid SCC (2/3, 66.6%) while low immunoexpression of EZH2 was predominantly found in keratinizing SCC (5/6, 83.3%) (Figure 1B) and in lymphoepithelioma-like carcinoma (1/1, 100%) (Table 2). Adenocarcinoma accounted for 8.33% (5/60) in our study (Table 2). The expression of EZH2 was variable in adenocarcinoma, depending upon the grade of tumor. Similar to our study, Liu et al. also studied 101 cases of different histologic subtypes of carcinoma cervix comprising of SCC (80/101), adenocarcinoma (17/101), and adenosquamous carcinoma (4/101). However, in contrast, they found no significant association between EZH2 expression and histologic subtypes (p = 0.855) [21]. Makk et al. also studied EZH2 expression in 37 cases of various histological types of adenocarcinoma and observed high expression of EZH2 in 98.14% of various adenocarcinomas and concluded that the robust EZH2 expression is an excellent diagnostic marker capable of differentiating the glandular neoplastic lesions from non-neoplastic lesions of cervix [9].

Majority of the cervical cancer in the present study were of Grade 2 (50/60, 83.4%) followed by Grade 1 (8/60, 13.3%) and Grade 3 (2/60, 3.3%) (Table 2). The EZH2 expression was observed to increase with increasing grades of carcinoma which was statistically significant (p = 0.001) (Table 2). Similar findings were reported by Liu et al. who studied 23 (23%) well-differentiated (Grade 1), 30 (30%) moderately differentiated (Grade 2), and 47 (47%) poorly differentiated (Grade 3) tumors and found that the tumor grade was significantly associated with EZH2 expression (p < 0.01) [21]. Thus, the immunoexpression of EZH2 can be analyzed in Grade 1 carcinomas and can be followed immunohistochemically to see any conversion to higher grade, as Grade 1 shows low expression and Grade 3 shows higher expression of EZH2. This can aid in monitoring the progression of Grade 1 carcinomas to higher grades which can further aid in deciding and reframing the treatment strategies according to the progression of EZH2 immunoexpression. However, Azizmohammadi et al. studied well / moderately differentiated (18/39, 46%) and poorly differentiated (21/39, 54%) cervical carcinomas and reported no significant correlation between EZH2 expression and different histological grades (p = 0.43) [24].

Out of 60 cases, lymphnode metastasis was histologically confirmed in 21 (35%) cases. Out of the 21 cases with lymph node metastasis, high EZH2 immunoexpression was found in 20 (20/21, 95.23%) cases and low immunoexpression in only one case (1/21, 4.7%) (Table 2). The association of EZH2 immunoexpression with lymphnode metastasis was statistically significant (p = 0.025) (Table 2). Similar findings were reported by Fatty et al. who found that 35% (19/54) cases of cervical cancer with lymph node metastasis had statistically significant association with EZH2 expression (p = 0.03) [6]. Similarly, Chen et al. studied EZH2 expressions in 168 patients with cervical SCC with 70 (42%) and 98 (58%) cases with and without lymph node metastasis respectively and obtained statistically significant correlation (p = 0.042) between EZH2 expression and lymph node metastasis [8]. Azizmohammadi et al. also analyzed EZH2 expression in 39 cases of carcinoma cervix with 14 (36%) and 25 (64%) cases with and without lymphnode metastasis and documented a that a statistically significant correlation (p < 0.05) exists between EZH2 expression and lymphnode metastasis [24]. However, in contrast to above studies, Jin et al. studied expression of EZH2 in 117 cases of cervical SCC and found no statistically significant correlation between EZH2 expression and lymphnode metastasis (p = 0.564) [22].

## Conclusions

The results of our study affirm that a significant association exists between immunohistochemical expression of EZH2 with tumor grade, histological subtype, lymphnode metastasis, and FIGO stage. The results of our study give way to future studies with larger sample size to further strengthen the association of EZH2 immunoexpression in cancer cervix patients which may aid in development of the targeted therapy in near future. Moreover, this study also affirms the use of IHC as robust, readily available technique to study the EZH2 immunoexpression.
